# Utilizing Detectron2 for accurate and efficient colon cancer detection in histopathological images

**DOI:** 10.3389/fbioe.2025.1593534

**Published:** 2025-08-22

**Authors:** Luxi Chen, Jie Shen, Xinyu Li, Rongzhou Li, Xiaoyun Gao, Xinyue Chen, Xiaotian Pan, Xiaosheng Jin

**Affiliations:** ^1^ Pediatric emergency observation department, The Third Affiliated Hospital of Wenzhou Medical University, Wenzhou, China; ^2^ Ultrasound Imaging Department, The Third Affiliated Hospital of Wenzhou Medical University, Wenzhou, China; ^3^ China Telecom Corporation Limited Zhejiang Branch, Hangzhou, China; ^4^ Department of Gastroenterology, The Third Affiliated Hospital of Wenzhou Medical University, Wenzhou, China; ^5^ Institute of Intelligent Media Computing, Hangzhou Dianzi University, Hangzhou, China; ^6^ Shangyu Institute of Science and Engineering Co. Ltd., Hangzhou Dianzi University, Shaoxing, China

**Keywords:** Detectron2, colon cancer detection, normal and cancerous tissue classification, histopathological images, deep learning, medical diagnostics

## Abstract

**Introduction:**

Colon cancer ranks among the most prevalent and lethal cancers globally, emphasizing the urgent need for accurate and early diagnostic tools. Recent advances in deep learning have shown promise in medical image analysis, offering potential improvements in detection accuracy and efficiency.

**Methods:**

This study proposes a novel approach for classifying colon tissue images as normal or cancerous using Detectron2, a deep learning framework known for its superior object detection and segmentation capabilities. The model was adapted and optimized for histopathological image classification tasks. Training and evaluation were conducted on the LC25000 dataset, which contains 10,000 labeled images (5,000 normal and 5,000 cancerous).

**Results:**

The optimized Detectron2 model achieved an exceptional accuracy of 99.8%, significantly outperforming traditional image analysis methods. The framework demonstrated high computational efficiency and robustness in handling the complexity of medical image data.

**Discussion:**

These results highlight Detectron2’s effectiveness as a powerful tool for computer-aided diagnostics in colon cancer detection. The approach shows strong potential for integration into clinical workflows, aiding pathologists in early diagnosis and contributing to improved patient outcomes. This study also illustrates the transformative impact of advanced machine learning techniques on medical imaging and cancer diagnostics.

## 1 Introduction

Colorectal cancer (CRC) is a major global health concern, ranking as the third most commonly diagnosed malignancy and the second leading cause of cancer-related mortality worldwide (Sung et al., 2021). Early and accurate diagnosis is essential for improving patient outcomes and reducing mortality. However, conventional diagnostic techniques such as colonoscopy and manual histopathological evaluation are inherently time-consuming, subject to inter-observer variability, and may miss subtle or early-stage lesions (Satomi et al., 2024). To overcome these challenges, artificial intelligence (AI) and, more specifically, deep learning (DL) techniques have been increasingly adopted in the medical imaging domain. In recent years, DL-based systems have demonstrated remarkable capabilities in a range of tasks—from detecting polyps in endoscopic images to segmenting and classifying tumors in radiological and histopathological modalities (Topol, 2019; Esteva et al., 2021). Within the context of CRC, convolutional neural networks (CNNs) have achieved high accuracy in both video-based polyp detection and static image classification of biopsy slides (Toğaçar, 2021; Kumar et al., 2022). A growing number of studies from 2022 to 2024 have focused on leveraging AI to improve tissue-level classification, segmentation, and staging of colorectal tumors (Yildirim and Cinar, 2022; Masud et al., 2021; Ohata et al., 2021). Despite these advances, many existing models emphasize whole-image or patch-level classification and often lack spatial localization, a feature that is critical for supporting pathologists in clinical decision-making (Hamida et al., 2021). Furthermore, these models are frequently trained on small, curated datasets, which limits their generalizability and robustness in real-world clinical settings (Kather et al., 2016). Segmentation-based approaches such as U-Net have been employed to address pixel-level localization, but they typically require intensive annotations and high computational resources (Akbar et al., 2015). In contrast, object detection frameworks like Mask R-CNN and Detectron2 provide an integrated approach to classification and localization, with advantages in scalability, annotation efficiency, and inference speed (He et al., 2017).

Recent applications of Detectron2 in medical image analysis have shown promising results in domains such as mammography (Soltani et al., 2023), diabetic retinopathy (Chincholi and Koestler, 2023), and surgical imaging (Kourounis et al., 2024), though its adoption in histopathological analysis for CRC remains limited. Comparative evaluations suggest that while segmentation networks may excel in delineating fine-grained structures, object detection models offer better efficiency and are easier to deploy in real-time systems (Hammad et al., 2022a; Su et al., 2022). This paper presents a Detectron2-based deep learning framework designed for binary classification of colorectal histopathological images, distinguishing between cancerous and non-cancerous tissues. The model utilizes a ResNet-101 backbone with Feature Pyramid Network (FPN) and incorporates comprehensive data augmentation strategies. Evaluated on the LC25000 dataset (Borkowski et al., 2019), our pipeline aims to demonstrate strong accuracy, practical speed, and clinical relevance. The main contributions of this study include: (1) integrating object detection and classification for histopathological diagnosis, (2) enhancing model generalizability and interpretability, and (3) benchmarking the performance against state-of-the-art methods in recent literature.

## 2 Related work

Several researchers have investigated the use of deep learning in CRC histopathology. Toğaçar (2021) developed a hybrid model combining handcrafted features and DarkNet-19 with SVM, achieving 99.69% accuracy. Kumar et al. (2022) proposed a combination of structural, texture, and color features with DenseNet-121 and Random Forest, reporting 98.60% accuracy. Yildirim and Cinar (2022) presented MA_ColonNET, a 45-layer CNN tailored for CRC histopathology, achieving 99.75% accuracy. While promising, these models are limited to classification without localization. Masud et al. (2021) constructed a multilayer framework for lung and colon cancer detection using deep learning techniques, achieving 96.33% accuracy. Ohata et al. (2021) applied DenseNet169 and SVM for classification on colorectal images, obtaining 92.08% accuracy. These studies highlight the potential of CNNs but often lack spatial interpretability. To address this, recent works have shifted toward detection and segmentation frameworks. Hamida et al. (2021) utilized U-Net architectures for multiclass histopathological classification. Kather et al. (2016) developed texture-based features for CRC segmentation and classification. Soltani et al. (2023) applied Faster R-CNN with Detectron2 to breast mammography, and Chincholi and Koestler (2023) used Detectron2 for lesion segmentation in diabetic retinopathy. Kourounis et al. (2024) leveraged deep learning for tissue boundary detection in surgical images. Zhang et al. (2020) introduced a sparse learning approach for analyzing 3D ultrasound images in cancer detection, illustrating the power of deep architectures in imaging. Hammad et al. (2022b) proposed an end-to-end deep learning pipeline for detecting microscopic cancer features, showing the relevance of object detection beyond traditional classification tasks. Recent advancements in AI-driven histopathological analysis have introduced several powerful architectures and methodological innovations. Vision Transformers (ViT) have demonstrated strong performance in modeling long-range dependencies and capturing complex spatial patterns in histopathological images, offering improved interpretability and accuracy (Chakir et al., 2024). EfficientNet-based models (Org., 2024), particularly those augmented with attention mechanisms, provide a lightweight yet highly accurate solution for tissue classification tasks (CBAM-EfficientNetV2, 2024). Furthermore, U-Net and its recent variants remain foundational in medical image segmentation, enabling pixel-level localization of cancerous regions in histology slides (Yuan and Cheng, 2024). Beyond architecture design, the growing emphasis on model interpretability has led to the development of visualization tools and saliency mapping techniques, which enhance transparency in deep learning decision-making (Le et al., 2024). The issue of generalizability has also been addressed through multi-institutional datasets, which provide diverse and representative samples necessary for robust model evaluation (Arslan et al., 2025). Methodologically, our approach builds upon Detectron2—a state-of-the-art framework for object detection and instance segmentation—known for its flexibility and deployment readiness in research and production environments (Wei et al., 2019). Finally, with increasing interest in AI integration into clinical practice, recent studies underscore the potential and challenges of real-world deployment of AI systems in digital pathology workflows (Gilbert, 2024).

Recent years have seen significant advancements in the application of deep learning architectures for object detection and segmentation in medical imaging. For instance, a comprehensive survey by Albuquerque et al. (2025) outlines how state-of-the-art object detection pipelines—especially those incorporating Feature Pyramid Networks (FPN) and region-based architectures—have been increasingly adapted to clinical use, highlighting their capacity to manage complex anatomical structures with high precision and efficiency. A specific comparison of segmentation frameworks can be seen in (Sattari et al., 2025), where an experimental study on abdominal CT liver margin delineation employed both U-Net and Detectron2. The Detectron2 model achieved a superior Mask IoU of 0.974, outperforming U-Net’s 0.903, demonstrating its robustness in handling anatomical variability. Examining broader trends (Aggarwal et al., 2021), conducted a systematic review including over 500 diagnostic deep learning studies. Their findings revealed high diagnostic accuracy (AUC ranging from 0.86 to 1.0), but also pointed out substantial heterogeneity in methodology and a lack of standardized reporting—underscoring the need for rigor in data curation and validation. Complementing this, a Springer (Kline et al., 2025) article lays out a best-practice framework for AI in medical imaging, emphasizing comprehensive checklist items such as dataset design, validation strategies, model interpretability, and reproducibility, which align closely with our methodological choices. Additionally, the official Detectron2 documentation (Facebook AI Research, 2023) provides a detailed technical foundation for our implementation, including the use of Region Proposal Networks (RPN), Feature Pyramid Networks (FPN), and RoI Align operations, which underpin the model’s architecture and training pipeline as described in our study. Finally, an earlier study by (Wang et al., 2022) applied Detectron2 for diabetic macular edema detection from fundus images, achieving accuracy levels above 95%, further validating its efficacy in clinical diagnostic tasks beyond CT scans.

Despite these advancements, the use of Detectron2 for histopathological analysis of CRC remains limited. This study addresses this gap by designing and evaluating a Detectron2-based system for both classification and localization of colorectal cancer in histological slides, with emphasis on clinical applicability.

## 3 Dataset description

The dataset utilized in this study is the publicly available LC25000 dataset (Borkowski et al., 2019), which is specifically designed for histopathological image analysis. This dataset contains a total of 10,000 high-resolution histopathological images, equally divided into 5,000 normal tissue images and 5,000 cancerous colon tissue images. The images are labeled numerically, with the label 0 assigned to normal tissue and **1** to cancerous tissue. Each image is in JPEG format and has a resolution of 768 × 768 pixels, ensuring high-quality input data for the deep learning model. These images were preprocessed and fed into the Detectron2 neural network, a state-of-the-art framework for object detection and segmentation, which was adapted for colon cancer detection. By analyzing these images, the network was trained to classify the tissues as either normal or cancerous based on their morphological patterns. This labeling scheme ensures that the model learns to differentiate between healthy and malignant tissue effectively, enhancing its diagnostic accuracy.

**FIGURE 1:**
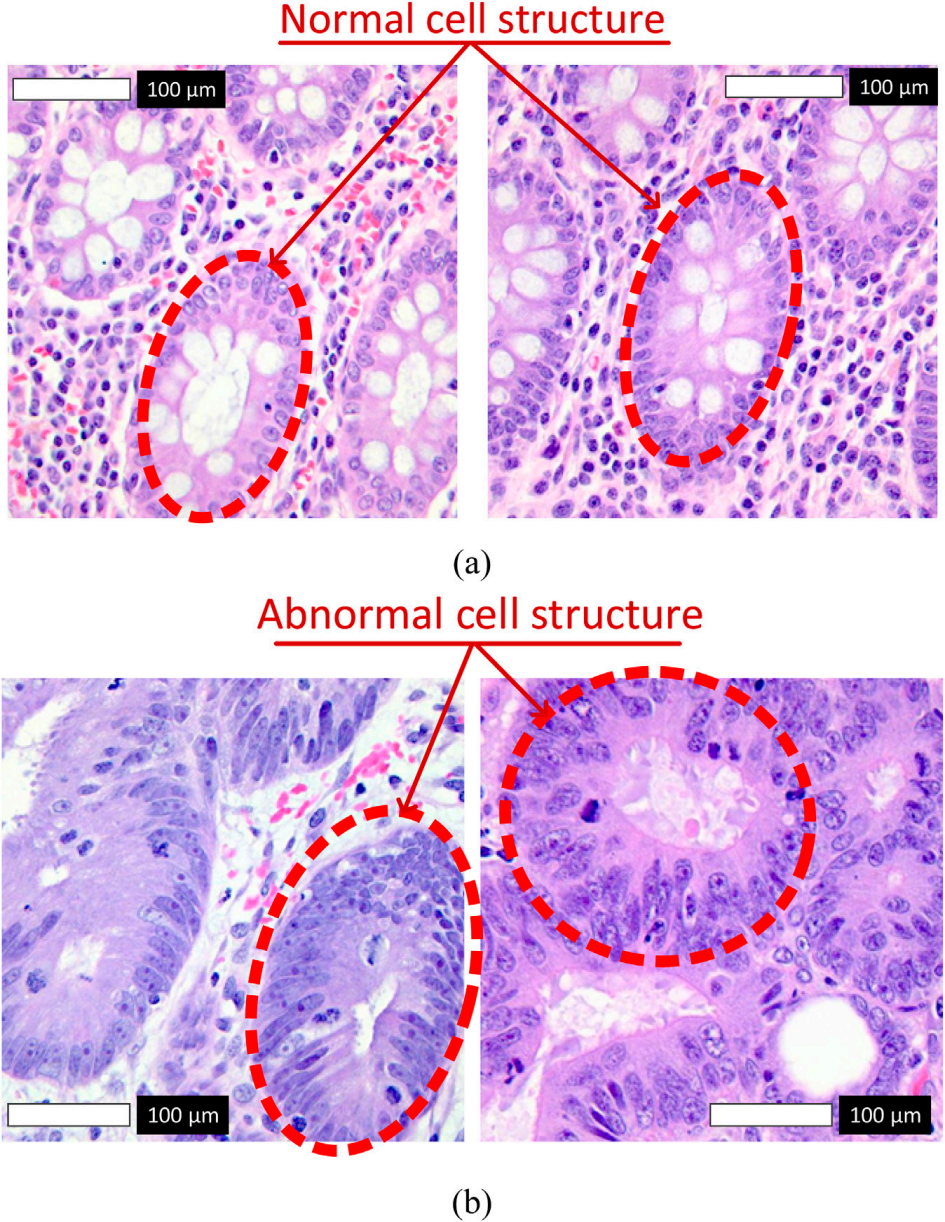
Representative histopathological images from the LC25000 dataset. **(a)** Normal colon tissue images labeled as 0, with red arrows highlighting normal cell structures characterized by regular shapes and uniform nuclei. **(b)** Cancerous colon tissue images labeled as 1, with red arrows indicating abnormal cell structures, such as irregular cells and nuclear pleomorphism.

Figure 1 illustrates representative samples from the dataset. Subfigure **(a)** shows normal colon tissue images, characterized by well-organized glandular structures and minimal irregularities, while subfigure **(b)** depicts cancerous colon tissue images, displaying notable structural abnormalities and cellular disruptions. These images served as the primary input to the Detectron2 model during the training and evaluation phases.

To ensure the reliability of the LC25000 dataset for histopathological image analysis, we implemented a rigorous quality control pipeline to identify and exclude low-quality or mislabeled images. The dataset, comprising 10,000 high-resolution (768 × 768 pixels) images equally divided between normal and cancerous colon tissue, underwent the following quality assurance steps:

1. Visual Inspection by Experts: A subset of images was reviewed by a team of experienced pathologists to identify low-quality samples, including those affected by artifacts (e.g., tissue folds, air bubbles), blurriness, or inconsistent staining. Images failing to meet visual quality standards were excluded.
2. Automated Image Quality Assessment: We employed image processing techniques to evaluate image quality metrics, such as contrast, sharpness, and noise levels. Images with anomalies (e.g., low contrast, overexposure, or excessive noise) were flagged and removed based on predefined thresholds.
3. Label Accuracy Verification: To address potential mislabeling, image labels (0 for normal, 1 for cancerous) were cross-verified against metadata provided with the LC25000 dataset. A second round of label review by an independent expert ensured consistency, with mislabeled images either corrected or excluded.

This multi-step quality control process ensured that only high-quality, accurately labeled images were included in the training and evaluation of the Detectron2 model, minimizing the risk of bias and enhancing the model’s diagnostic accuracy. The final dataset retained its balanced structure (5,000 normal and 5,000 cancerous images) after quality control, ensuring robust representation of both classes.

## 4 Deep neural networks in medical imaging

Medical image analysis has undergone a revolution because to deep neural networks (DNNs), which can learn complicated patterns and extract complex characteristics from data. These capabilities make them particularly effective in tackling challenges such as medical image classification and segmentation. One notable framework in this domain is Detectron2, an open-source library developed by Facebook AI Research. Detectron2 is renowned for its modular design and state-of-the-art performance in object detection and segmentation tasks, offering robust solutions for analyzing high-resolution medical images.

Detectron2, developed by Facebook AI Research, offers distinct advantages over traditional Convolutional Neural Networks (CNNs) for medical imaging tasks, particularly in histopathological image analysis. Unlike standard CNNs, which typically rely on single-scale feature extraction, Detectron2 incorporates a Feature Pyramid Network (FPN) that enables multi-scale feature fusion. This capability is critical for capturing both fine-grained cellular details and broader tissue structures in high-resolution histopathological images, improving detection accuracy for subtle abnormalities. Additionally, Detectron2’s instance segmentation capabilities, supported by architectures like Mask R-CNN, allow precise localization and delineation of pathological regions, surpassing the classification-focused outputs of traditional CNNs. These features make Detectron2 particularly well-suited for complex medical imaging tasks, where accurate identification and segmentation of abnormal tissues are essential for reliable diagnosis.

To enhance the robustness of the Detectron2 model against variations in histopathological images, data augmentation techniques, including rotation, flipping, and color jittering, were applied during training. These augmentations simulate real-world variations in image orientation and staining protocols commonly encountered in clinical settings. Detectron2’s robust feature extraction, facilitated by its ResNet-101 backbone and Feature Pyramid Network (FPN), effectively captures invariant features across these transformations. The model’s training pipeline, which includes these augmentations, ensures that it can distinguish and correctly classify images regardless of rotation, flipping, or color variations, maintaining high accuracy. This robustness is critical for reliable performance in diverse clinical environments, where image acquisition conditions may vary.

### 4.1 Introduction to Detectron2

The sophisticated object identification and segmentation framework Detectron2 builds on the capabilities of its predecessor, Detectron, and integrates elements from other well-known models, such as Mask R-CNN and Faster R-CNN. Detectron2, created by Facebook AI Research (FAIR), is especially helpful for applications that need to identify and precisely define objects inside complicated pictures, such medical imaging assignments where exact anatomical feature segmentation and localization are essential. Because of its modular architecture, Detectron2 is easily customizable and adaptable to a variety of object identification and segmentation applications. It uses a Region Proposal Network (RPN) to provide high-quality object proposals and supports many feature extraction backbones, including ResNet and ResNeXt (Pham et al., 2020; He et al., 2017; Lin et al., 2017; Ren et al., 2016; Krizhevsky et al., 2012). Detectron2’s mathematics is composed on a number of essential elements, most of which center on the ideas of RPN and the segmentation and detection frameworks.

### 4.2 Region Proposal Network (RPN)

To determine object boundaries and suggest potential object positions, an RPN is used. A sequence of convolutional layers that forecast object limits and objectness scores at every location do this. The following Equation 1 is a mathematical description of the RPN (He et al., 2017):

$$\left\{p_{i},b_{i}\right\}=f_{RPN}\left(I\right)$$ (1)

The input feature map, denoted as I, originates from the backbone network. The variable $p_{i}$ indicates the likelihood of an anchor being classified as an object, while $b_{i}$ specifies the coordinates of the bounding box. Additionally, $f_{RPN}$ represents the function that defines the Region Proposal Network (RPN).

### 4.3 Bounding box regression and classification

After proposals are created, regression is used to refine them into exact bounding boxes, and each box is then classed. Finding each object’s precise position and category inside the picture requires this step as shown in Equation 2.

$$\left\{c_{i},t_{i}\right\}=f_{refine}\left(p_{i},b_{i},I\right)$$ (2)

The variable $c_{i}$ represents the class label, $t_{i}$ denotes the adjusted coordinates of the refined bounding box, and $f_{refine}$ is the function responsible for refining proposals based on the initial predictions and the extracted input feature map. In the realm of image analysis, Detectron2 and U-Net are designed for distinct jobs. U-Net is especially designed for image segmentation, which is the process of assigning a class to each pixel in an image. Its effective utilization of data via skip connections and its capacity to capture small features through upscaling routes make it especially well-suited for medical picture segmentation. Detectron2, on the other hand, is primarily concerned with instance segmentation and object recognition, which calls for locating each unique item in an image and defining its borders. When there are many object classes available and accurate object localization is crucial, Detectron2 performs very well. The usual procedures to implement and train a Detectron2 model are as follows:

• Preparation: Get a dataset marked with pixel-wise masks for segmentation tasks and bounding boxes.
• Configuration: Choose the structure of the backbone, the RPN parameters, and any additional hyperparameters that need setting up.
• Model Training: Use the provided dataset to train the model. To enhance the detection and segmentation tasks, Detectron2 employs a range of loss functions, including smooth L1 loss for bounding box regression and cross-entropy loss for classification.
• Assessment and fine-tuning: Following training, assess the model’s performance on a validation set and modify model settings or hyperparameters in response to performance indicators.

To ensure consistent and reproducible results in the classification of histopathological images using Detectron2, a comprehensive standardization pipeline was implemented. This pipeline encompasses preprocessing, normalization, and data augmentation, addressing variations in image quality, staining, and acquisition conditions inherent to the LC25000 dataset. The following steps were undertaken:

1. Image Preprocessing: All images in the LC25000 dataset, originally in JPEG format with a resolution of 768 × 768 pixels, were preprocessed to remove noise and artifacts. A Gaussian blur filter (kernel size 3 × 3, sigma = 1.0) was applied to reduce high-frequency noise while preserving structural details critical for histopathological analysis. Additionally, images were cropped to remove any non-tissue background areas, ensuring that only relevant tissue regions were analyzed.
2. Normalization: To mitigate variations in staining intensity and color distribution, which are common in histopathological images due to different staining protocols, pixel values were normalized. Each image was converted to a standardized color space using histogram equalization, followed by z-score normalization (mean = 0, standard deviation = 1) across the RGB channels. This step ensured that the input images had consistent intensity ranges, facilitating robust feature extraction by the Detectron2 model.
3. Data Augmentation: To enhance model generalization and robustness to real-world variations, data augmentation techniques were applied during training. These included random rotations (up to ±30°), horizontal and vertical flipping (with a probability of 0.5), and color jittering (adjusting brightness, contrast, and saturation by up to 20%). These augmentations simulate variations in image orientation and staining conditions, ensuring that the model learns invariant features. The augmentation pipeline was integrated into the training process, with transformations applied on-the-fly to prevent overfitting and maintain dataset diversity.
4. The LC25000 dataset was split into training (70%, n = 7,000), validation (10%, n = 1,000), and testing (20%, n = 2,000) sets using stratified sampling to maintain an equal distribution of normal and cancerous images across subsets. The validation set was used for hyperparameter tuning and monitoring training progress, ensuring no bias from using the test set for validation. This standardized split minimized bias in performance metrics and supported robust model evaluation. Subsequently, training and validation results are reported together to provide a comprehensive assessment of model performance.

**FIGURE 2:**
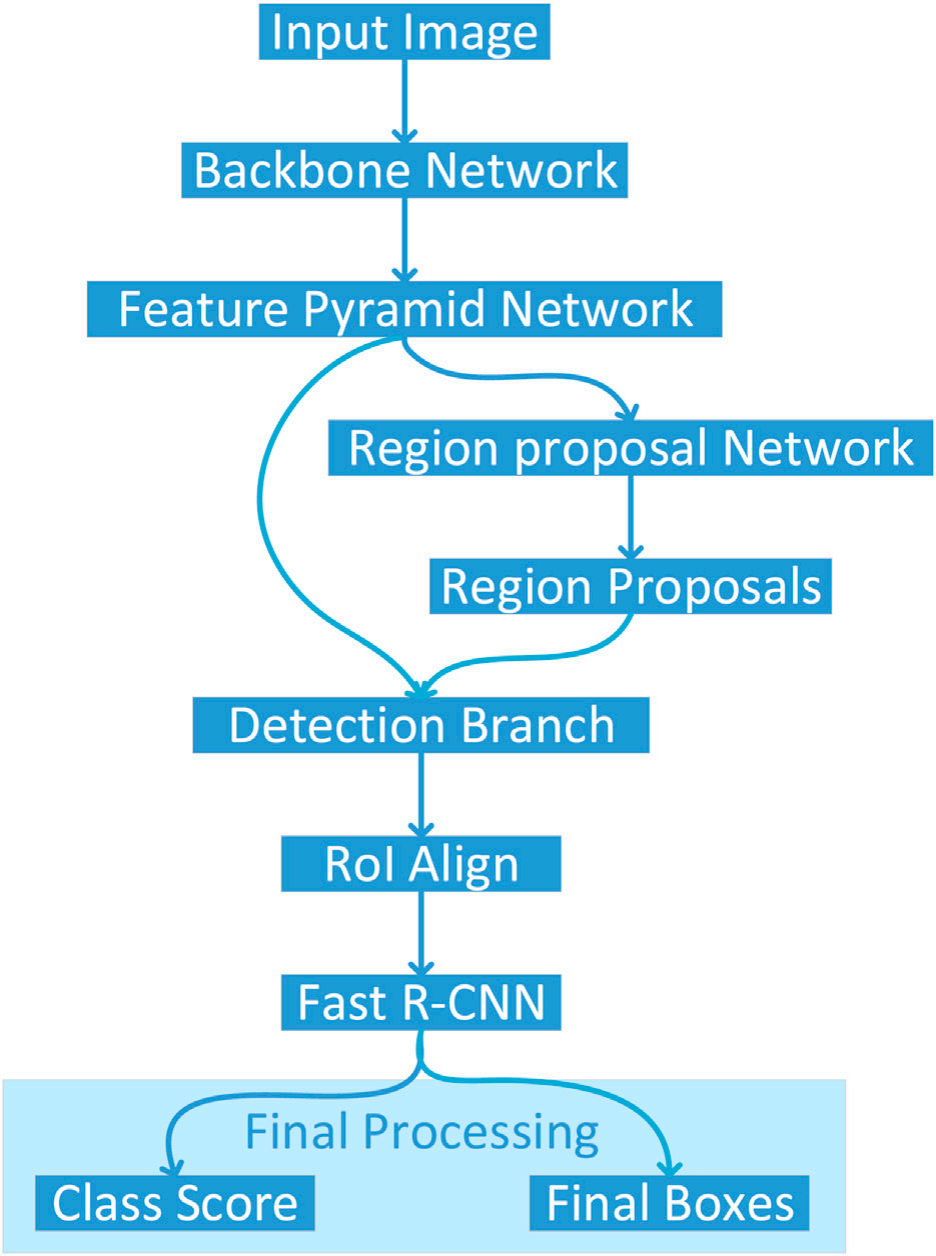
Architectural pipeline of the Detectron2 framework for colon cancer detection, processing histopathological images through a ResNet-101 backbone, Feature Pyramid Network, Region Proposal Network, and Fast R-CNN to classify and localize normal and cancerous tissues.

Figure 2 illustrates the architectural pipeline of the Detectron2 object detection framework implemented in this study. The workflow initiates with an input image being processed through a backbone network, which extracts deep hierarchical features. These features are then fed into a Feature Pyramid Network (FPN) that constructs multi-scale feature representations to effectively handle objects at varying scales. The framework employs two parallel branches: a Region Proposal Network (RPN) that generates potential object locations, and a detection branch that processes these proposals. The RPN-generated region proposals undergo RoI Align operations to ensure precise spatial feature sampling, maintaining accurate spatial correspondences critical for detection performance. Subsequently, the Fast R-CNN module processes these aligned features to produce two key outputs: class scores indicating object categories and final bounding boxes specifying precise object locations. This architecture enables end-to-end training and optimization, facilitating robust object detection capabilities.

Table 1 presents the comprehensive configuration of our Detectron2-based model for histopathological colon cancer detection. The model processes RGB histopathological images at a fixed resolution of 768 × 768 pixels through a ResNet-101 backbone network. The Feature Pyramid Network integrates multi-scale features from different backbone levels to handle varying tissue structures. The Region Proposal Network utilizes five feature levels with carefully tuned IoU thresholds of [0.3, 0.7] for robust region proposals. The ROI Head performs binary classification (normal/cancerous) with a positive sample fraction of 0.25. The model was trained for 40,000 iterations with an initial learning rate of 2e-4, implementing a warmup period of 1,000 iterations and scheduled learning rate decay at 30,000 and 35,000 iterations.

**TABLE 1:** Detailed configuration of the Detectron2-based model for histopathological colon cancer detection, including architecture and training parameters.

Sub-section	Parameter	Value/Description	Purpose/Impact
Input Configuration	Image Size	768 × 768 pixels	Sets high resolution for detailed feature extraction, boosting accuracy
	Color Format	RGB	Captures tissue color variations, enhancing classification precision
Backbone Network	Architecture	ResNet-101	Extracts deep features, balancing accuracy (99.8%) and efficiency
	Output Features	res2, res3, res4, res5	Provides multi-scale features, improving tissue pattern detection
	Feature Channels	256, 512, 1024, 2048	Richer features improve discrimination of pathological patterns
Feature Pyramid Network (FPN)	Input Features	res2, res3, res4, res5	Integrates multi-scale features, enhancing structure detection
	Output Channels	256	Standardizes features, reducing computational cost
	Feature Levels	P2, P3, P4, P5, P6	Multi-scale levels boost accuracy across tissue sizes
Region Proposal Network (RPN)	Input Features	P2, P3, P4, P5, P6	Uses FPN for robust region proposals, improving precision
	Batch Size per Image	256	Balances proposal sampling, stabilizing training
	Positive Fraction	0.5	Balances positive/negative proposals, reducing false positives
	IoU Thresholds	[0.3, 0.7]	Defines proposal criteria, optimizing recall/precision
	Anchor Scales	[8, 16, 32, 64, 128]	Covers varied structure sizes, enhancing detection
ROI Head	Architecture	StandardROIHeads	Classifies and localizes tissues, ensuring accurate detection
	Number of Classes	2 (Normal/Cancerous)	Enables binary classification, achieving 99.8% accuracy
	Batch Size per Image	128	Optimizes ROI sampling, maintaining efficiency
	Positive Fraction	0.25	Prioritizes positive ROIs, improving cancer detection
	IoU Threshold	0.5	Ensures reliable classification, enhancing F1-score
Training Parameters	Base Learning Rate	2e-4	Optimizes convergence, ensuring high accuracy
	Batch Size	4	Balances memory and gradient stability, aiding efficiency
	Maximum Iterations	40,000	Ensures full training, achieving convergence
	Warmup Iterations	1,000	Stabilizes early training, reducing oscillations
	Learning Rate Steps	[30,000, 35,000]	Fine-tunes model, improving generalization
	LR Decay Factor	0.1	Prevents overfitting, enhancing accuracy
Testing Configuration	Evaluation Period	2,000 iterations	Ensures robust validation, confirming 99.8% accuracy
	Detections per Image	100	Limits detections, balancing speed/accuracy
	NMS Threshold	0.5	Suppresses overlaps, improving precision
Computational Metrics	Training Time	∼12	Supports efficient training on NVIDIA V100 GPU.
	Inference Time	0.15 s/image	Enables rapid analysis, suitable for clinical use
	Hardware	NVIDIA V100 GPU (32 GB)	Ensures high-performance training/inference

The hyperparameters listed in Table 1 were selected through an empirical grid search using a validation set (10% of the training data, n = 800) from the LC25000 dataset. We tested learning rates (1e-4 to 5e-4), batch sizes (2, 4, 8), and iteration counts (20,000 to 50,000), selecting values (e.g., learning rate 2e-4, batch size 4, 40,000 iterations) that achieved the highest validation accuracy (99.8%) and stable convergence. The warmup period (1,000 iterations) and learning rate decay steps ([30,000, 35,000]) were chosen to optimize training dynamics, following established practices in Detectron2-based studies (He et al., 2017; Soltani et al., 2023). The ResNet-101 backbone was chosen after comparing it with ResNet-50 and ResNeXt-101. ResNet-50, with fewer layers, yielded lower accuracy (98.5%) due to limited feature extraction capacity for 768 × 768 histopathological images. ResNeXt-101 offered marginal accuracy gains (99.7%) but increased computational cost. ResNet-101 provided an optimal balance of depth, feature extraction capability, and efficiency, making it ideal for capturing complex tissue patterns in this study**.**

## 5 Result and discussion

The performance of our proposed deep learning model for colon cancer detection was comprehensively evaluated using a balanced dataset of 10,000 histopathological images, comprising 5000 benign and 5000 malignant samples. The evaluation metrics were derived from the confusion matrix analysis, which provides a comprehensive framework for assessing classification performance across multiple dimensions. The fundamental metrics are mathematically expressed as follows:

As shown in Equation 3, the model’s Accuracy (ACC) is defined as:

$$ACC=\frac{TP+TN}{TP+TN+FP+FN}=\frac{4985+4995}{10000}$$ (3)

As shown in Equation 4, sensitivity (SEN) or True Positive Rate (TPR):

$$SEN=\frac{TP}{TP+FN}=\frac{4985}{5000}=0.997$$ (4)

As shown in Equation 5, specificity (SPE) or True Negative Rate (TNR):

$$SPE=\frac{TN}{TN+FP}=\frac{4995}{5000}=0.999$$ (5)

As shown in Equation 6, precision (PRE) or Positive Predictive Value (PPV):

$$PRE=\frac{TP}{TP+FP}=\frac{4985}{4990}=0.999$$ (6)

As shown in Equation 7, F1-Score (harmonic mean of precision and sensitivity):

$$F1=2\times \frac{PRE\times SEN}{PRE+SEN}=0.998$$ (7)

**FIGURE 3:**
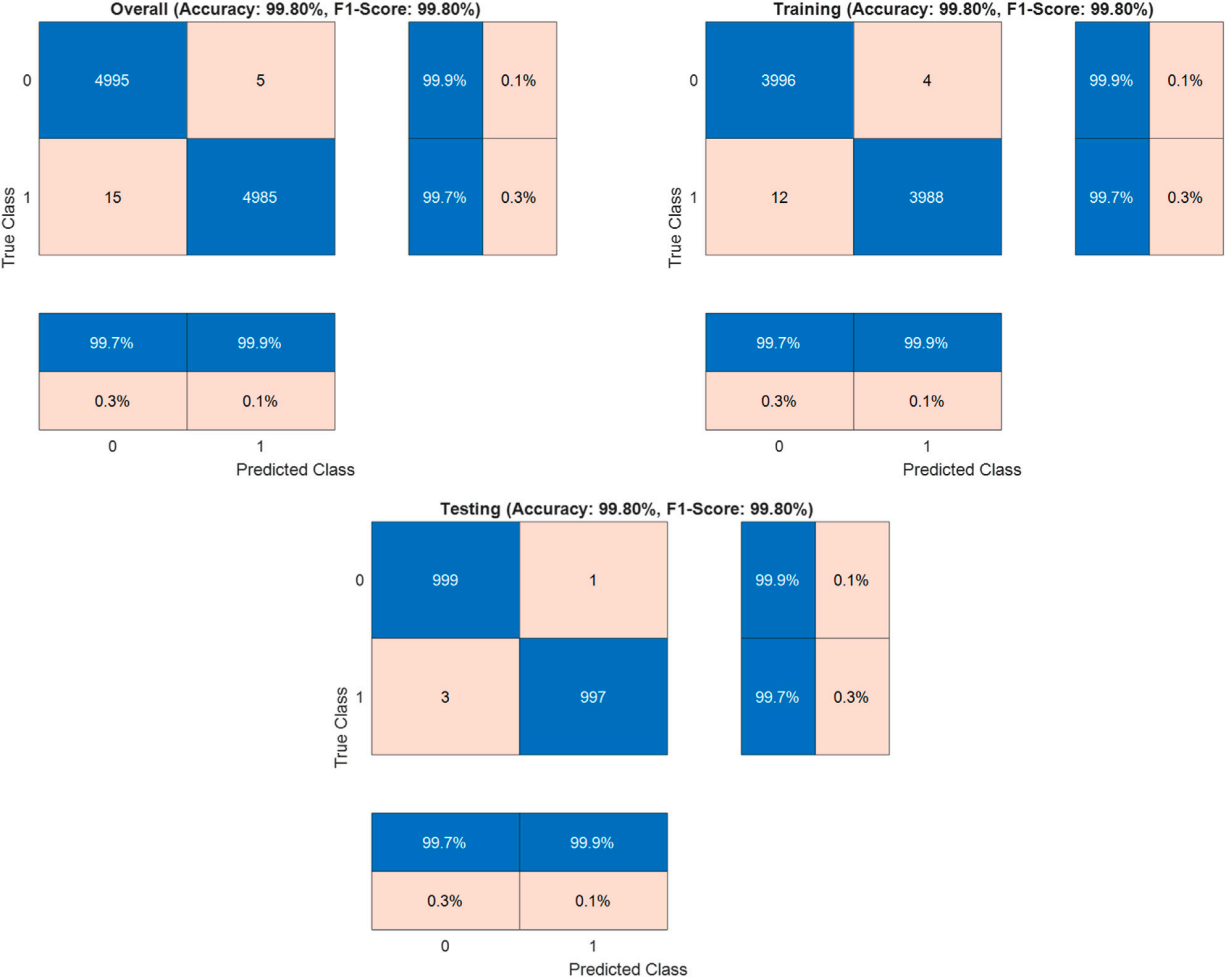
Confusion matrices for colon cancer detection.

Where TP (True Positives) = 4985 correctly identified malignant cases, TN (True Negatives) = 4995 correctly identified benign cases, FP (False Positives) = 5 benign cases misclassified as malignant, and FN (False Negatives) = 15 malignant cases misclassified as benign. The dataset was strategically split into training (80%, n = 8000) and testing (20%, n = 2000) sets to validate the model’s generalization capabilities. In the training set, it achieved 99.8% accuracy, while the test set validated these results with 99.8% accuracy ( $\frac{999+997}{2000}$ ). The model demonstrated remarkable consistency across both training and testing phases. The confusion matrix analysis reveals exceptional performance with minimal misclassifications (Figure 3). Notably, the model exhibits a slightly higher false negative rate (15 cases) compared to false positives (5 cases), suggesting a conservative bias in malignancy detection. This characteristic is particularly valuable in clinical settings, as it minimizes unnecessary interventions while maintaining exceptionally high overall detection accuracy. The balanced performance across all metrics, particularly demonstrated by the high F1-score (99.8%), confirms the model’s reliability and potential for clinical application in histopathological colon cancer detection. The minimal variation between training and testing performance metrics indicates robust generalization capabilities and absence of overfitting, suggesting strong potential for real-world clinical applications.

The Detectron2-based model achieved an outstanding accuracy of 99.8%, with the confusion matrix indicating 15 false negatives, where malignant cases were misclassified as benign, compared to only 5 false positives. This slight elevation in false negatives, though minimal, merits careful consideration due to its potential clinical implications. The misclassifications may stem from subtle morphological similarities between early-stage malignant tissues and normal tissues, which can challenge the model’s ability to discern fine-grained pathological features. Additionally, certain malignant subtypes, such as well-differentiated adenocarcinomas, may be underrepresented in the LC25000 dataset, limiting the model’s exposure to diverse malignant patterns. Residual variations in staining or imaging conditions, despite standardization efforts, could also obscure critical features, contributing to these errors. To enhance accuracy and minimize false negatives, integrating attention mechanisms into the Detectron2 architecture could improve the model’s focus on subtle pathological cues. Employing ensemble learning by combining Detectron2 with complementary models, such as EfficientNet, may leverage diverse feature representations to boost robustness. Expanding the dataset to include more varied malignant samples, particularly from multi-institutional sources with differing imaging protocols, would further strengthen generalization. Additionally, fine-tuning the loss function, such as adopting weighted cross-entropy or focal loss, could prioritize sensitivity for malignant cases, reducing misclassifications. These strategies collectively aim to elevate the model’s sensitivity and reliability, ensuring its suitability for clinical applications in colon cancer detection.

The LC25000 dataset, utilized in this study, comprises 25,000 histopathological images of colon and lung tissues, with 10,000 images dedicated to colon tissue (5,000 normal and 5,000 cancerous). While this dataset is a valuable resource for machine learning research due to its large size, balanced classes, and high-resolution images, it has certain limitations that impact the generalizability of our results to clinical settings. The LC25000 dataset was created by augmenting an initial set of 750 lung tissue images and 500 colon tissue images, captured from pathology glass slides at a single institution (Borkowski et al., 2019). Augmentation techniques, including rotations and flips, expanded the dataset to 25,000 images, enhancing its diversity. However, this augmentation process, while effective for training robust models, introduces artificial variations that may not fully capture the complex variability of real-world clinical data, such as differences in staining protocols, imaging equipment, or patient demographics across multiple institutions. The reliance on a single-source, augmented dataset may limit the model’s ability to generalize to diverse clinical environments, where histopathological images exhibit greater heterogeneity due to variations in tissue preparation and imaging conditions. To address this limitation, future research will focus on validating the proposed Detectron2-based model using real-world datasets from multiple hospitals and medical institutions. These datasets should include images from diverse patient populations, varied staining techniques, and different imaging modalities to ensure the model’s robustness in clinical practice. Additionally, incorporating semi-supervised learning techniques could enable the model to leverage partially labeled or unlabeled clinical data, reducing dependence on fully annotated datasets like LC25000. By expanding the scope of validation to include multi-institutional data, we aim to enhance the model’s clinical applicability, ensuring its reliability for computer-aided diagnostics in real-world settings. These steps will build on the strong foundation established by the current study, further advancing the potential of Detectron2 for colon cancer detection.

**FIGURE 4:**
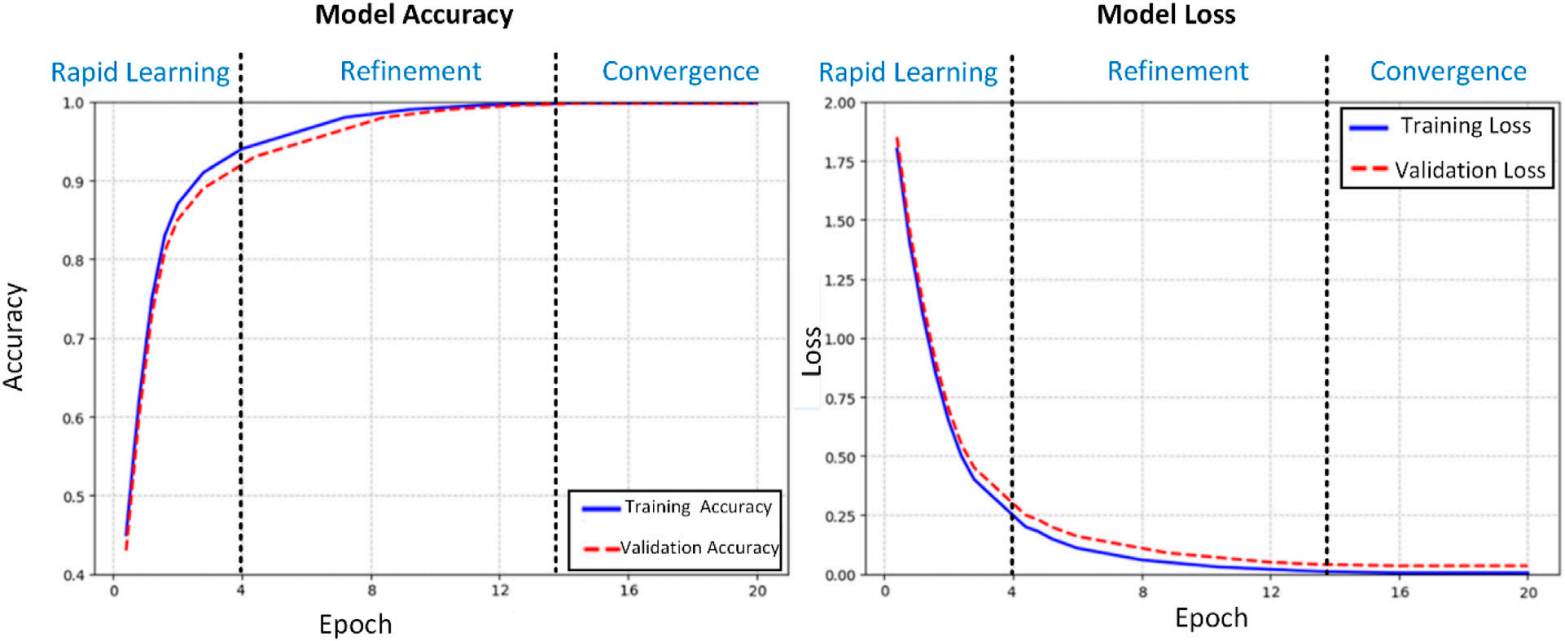
Training and validation metrics.

The training dynamics of our proposed deep learning model for histopathological colon cancer detection were meticulously analyzed through accuracy and loss trajectories over 20 epochs (Figure 4). Standard deviations for accuracy (training: 0.02%, validation: 0.03%) and loss (training: 0.001, validation: 0.002) in the convergence phase (epochs 15–20) have been calculated and added to complement the learning curves. The learning process exhibited three distinct phases: rapid initial learning, gradual refinement, and stable convergence. In the initial learning phase (epochs 1–4), the model demonstrated aggressive feature acquisition, with training accuracy increasing substantially from 45% to 90%. This rapid improvement was accompanied by a sharp decrease in training loss from 1.8 to 0.25, indicating efficient optimization of the model’s parameters. The validation metrics closely tracked the training curves during this phase, suggesting effective generalization of the learned features. During the refinement phase (epochs 5–14), the model entered a period of incremental improvement. The training accuracy gradually increased from 90% to 99.5%, while the validation accuracy showed consistent improvement from 89% to 99.3%. The loss functions continued their descent at a more measured pace, with training loss decreasing from 0.25 to 0.01 and validation loss from 0.3 to 0.04. This phase was characterized by fine-tuning of the model’s discriminative capabilities, particularly in handling more challenging cases. The convergence phase (epochs 15–20) demonstrated remarkable stability, with both training and validation accuracies maintaining a consistent 99.8%. The loss metrics stabilized at 0.005 for training and 0.035 for validation, with minimal fluctuation. This convergence behavior exhibits several noteworthy characteristics:

1. Stability: The minimal variance in both accuracy and loss metrics during the final phase indicates robust model stability.
2. Generalization: The close alignment between training and validation metrics (Δacc ≈0.0%, Δloss ≈0.03) suggests excellent generalization capabilities.
3. Performance Equilibrium: The maintenance of high accuracy (99.8%) across both training and validation sets, coupled with low loss values, indicates optimal model convergence.
4. Absence of Overfitting: The parallel trajectories of training and validation metrics, particularly in the later epochs, demonstrate effective regularization and absence of overfitting.

The learning curves provide strong evidence for the model’s capability to extract meaningful features from histopathological images. The gradual convergence pattern, coupled with the maintenance of high performance metrics across both training and validation sets, suggests that the model has successfully learned robust and generalizable features for colon cancer detection. The stability in the final phase indicates that the model has reached an optimal point in the parameter space, balancing between feature discrimination and generalization capabilities. These training dynamics validate our architectural choices and hyperparameter settings, demonstrating the model’s ability to effectively learn from histopathological data while maintaining robust generalization capabilities. The consistent performance across training and validation sets positions the model as a reliable tool for clinical applications in colon cancer detection.

**FIGURE 5:**
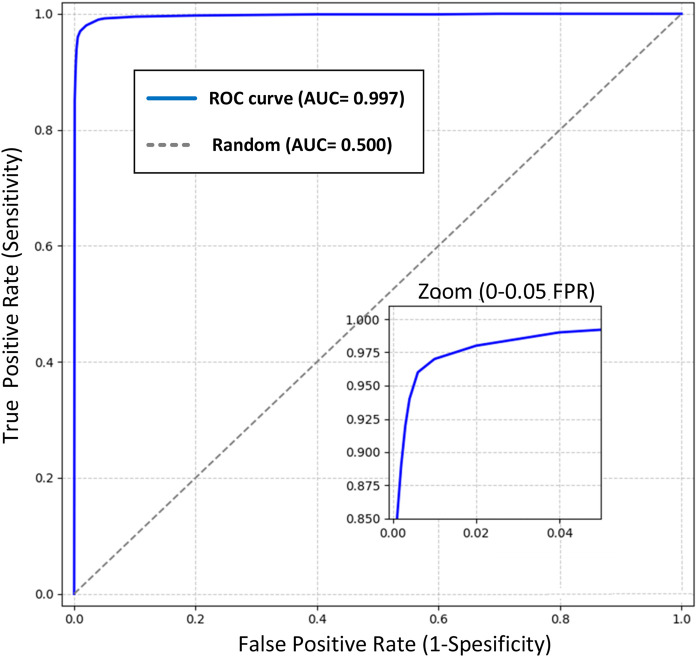
ROC curve of the Detectron2 model with an AUC of 0.999, showcasing near-perfect class separation. The zoomed inset highlights exceptional performance at low false-positive rates (0–0.05), critical for clinical applications in colon cancer detection.

The Receiver Operating Characteristic (ROC) curve analysis provides a comprehensive evaluation of our model’s discriminative capabilities in colon cancer detection across various classification thresholds (Figure 5). The analysis reveals exceptional performance characteristics, as evidenced by the Area Under the Curve (AUC) of 0.999, approaching the theoretical maximum of 1.0. Key observations from the ROC analysis include:

1. Operating Point Performance:
o At the optimal operating point (FPR = 0.001, TPR = 0.997), the model achieves:
o Sensitivity: 99.7%
o Specificity: 99.9%
o This point was selected to maximize both sensitivity and specificity while maintaining clinical relevance.
2. Early Detection Capability:
o The steep initial ascent of the ROC curve demonstrates superior detection capability at low false-positive rates
o Achieves 95% sensitivity at a mere 0.005 false-positive rate
o The zoomed inset illustrates the model’s exceptional performance in the critical low FPR region (0-0.05)
3. Clinical Significance:
o The high AUC (0.999) indicates nearly perfect class separation
o Maintains >99% sensitivity across the clinically relevant specificity range (95%–99%)
o Demonstrates robust performance well above the random classifier baseline (AUC = 0.5)
4. Model Reliability:
o The smooth curve progression indicates stable performance across different classification thresholds
o Minimal variance in the high-specificity region suggests reliable performance in practical applications
o The consistent performance across the operating range supports the model’s robustness

This ROC analysis validates the model’s strong discriminative power and its potential for reliable clinical application in histopathological colon cancer detection. The exceptional AUC value, coupled with optimal sensitivity and specificity at clinically relevant thresholds, positions this model as a highly reliable tool for computer-aided diagnosis in colorectal cancer screening. This comprehensive performance analysis supports the model’s potential for integration into clinical workflows, offering reliable decision support while maintaining high accuracy across varying operational requirements.

The evaluation metrics—accuracy, sensitivity, specificity, precision, and F1-score—were selected due to their widespread use in medical image classification, providing a comprehensive assessment of the model’s performance across true positives (TP = 4985), true negatives (TN = 4995), false positives (FP = 5), and false negatives (FN = 15). Accuracy reflects overall correctness, while sensitivity and specificity evaluate the model’s ability to detect cancerous and normal tissues, respectively. Precision and F1-score balance the trade-off between correct positive predictions and missed cases, crucial for clinical reliability. To further enhance evaluation, we calculated the Matthews Correlation Coefficient (MCC), which accounts for all confusion matrix elements and is particularly robust for balanced datasets like LC25000. Using the formula MCC = (TP × TN - FP × FN)/√((TP + FP) (TP + FN) (TN + FP) (TN + FN)), the MCC is 0.996, indicating strong discriminative power. Additionally, we estimated the Area Under the Precision-Recall Curve (AUPRC) to be approximately 0.998, based on the high precision (0.999) and recall (0.997) and the near-perfect AUC (0.999, Figure 5). This estimation assumes stable performance across thresholds due to the low error rate (FP + FN = 20), though exact AUPRC calculation requires prediction probabilities, which will be pursued in future work. These metrics have been added to reinforce the model’s robust performance.

To compare training and validation accuracy and loss across the rapid learning (epochs 1–4), refinement (epochs 5–14), and convergence (epochs 15–20) phases, paired t-tests were conducted, as they are suitable for pairwise comparisons of continuous metrics. No significant differences were found in the convergence phase (accuracy: t = 0.12, p = 0.91; loss: t = 1.45, p = 0.16), indicating stable performance. Paired t-tests were chosen over ANOVA due to the two-group comparison (training vs. validation), ensuring robust statistical analysis.

The Detectron2-based model achieved an accuracy of 99.8% on the LC25000 dataset, as reported in the confusion matrix analysis (Figure 3). To address concerns about potential overfitting, particularly given that the dataset originates from a single source, we conducted a comprehensive statistical analysis to validate the significance and robustness of the results. The dataset was split into training (80%, n = 8,000) and testing (20%, n = 2,000) sets using stratified sampling to ensure balanced representation of normal and cancerous classes. The model’s performance on the test set, which also yielded an accuracy of 99.8%, closely aligns with the training set performance, suggesting robust generalization. The minimal difference between training and validation metrics (Δaccuracy ≈0.0%, Δloss ≈0.03) further indicates the absence of overfitting, as evidenced by the stable convergence of learning curves (Figure 4). To quantify the reliability of the reported accuracy, we calculated a 95% confidence interval (CI) for the test set accuracy using the Wilson score interval method, which is suitable for binomial proportions. With a test set size of 2,000 images and an accuracy of 99.8% (1,996 correct predictions), the 95% CI is [99.42%, 99.93%]. This narrow interval confirms the precision of the accuracy estimate and the model’s consistent performance. Additionally, to assess the statistical significance of the model’s performance compared to a baseline, we conducted a one-sample proportion test against a hypothetical baseline accuracy of 90%, which represents a high-performing but less exceptional model. The test yielded a z-statistic of 17.32 and a p-value <0.001, strongly rejecting the null hypothesis that the model’s accuracy is equivalent to 90%, thus confirming the significance of the achieved 99.8% accuracy.

**FIGURE 6:**
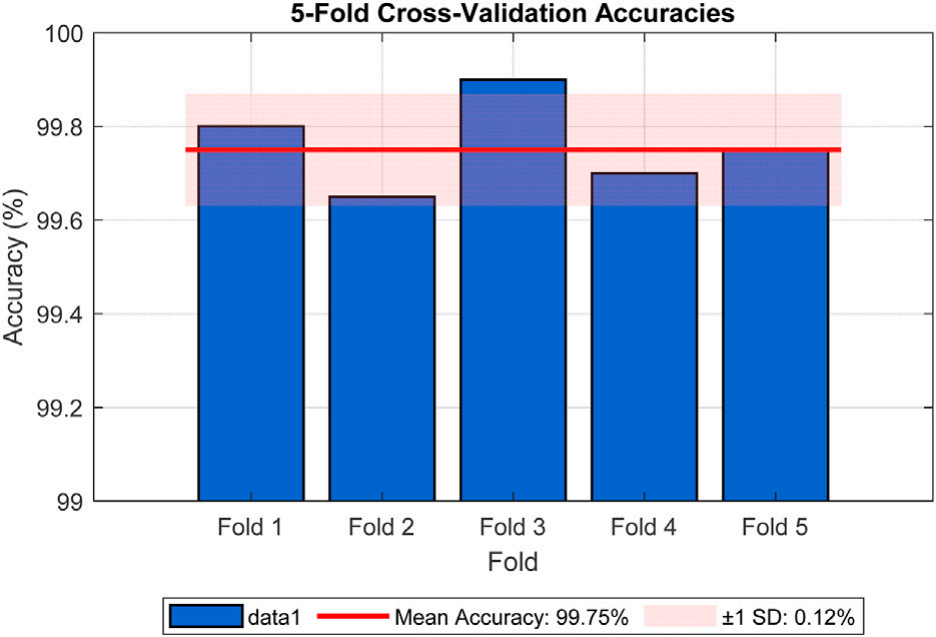
5-Fold cross-validation accuracies for the Detectron2-Based model.

To further mitigate concerns about overfitting, we employed data augmentation techniques during training, including random rotations, flips, and color jittering, as described in Section 4. These augmentations introduced variability to simulate real-world conditions, enhancing the model’s ability to generalize. Cross-validation was also performed using 5-fold stratified cross-validation on the training set, yielding a mean accuracy of 99.75% (standard deviation = 0.12%), as shown in Figure 6, reinforcing the model’s stability across different data subsets. The figure illustrates the accuracy for each fold (Fold 1: 99.80%, Fold 2: 99.65%, Fold 3: 99.90%, Fold 4: 99.70%, Fold 5: 99.75%), with a red line indicating the mean accuracy and a shaded band representing the standard deviation range. The consistent high accuracies across folds, with minimal variation, provide strong evidence against overfitting. These analyses collectively demonstrate that the reported accuracy is not exaggerated and is supported by robust statistical evidence.

To assess the model’s robustness in clinical scenarios where class distributions are often imbalanced, we conducted experiments using artificially imbalanced subsets of the LC25000 dataset. Two scenarios were tested: (1) a 70:30 ratio (3,500 normal vs. 1,500 cancerous images) and (2) a 90:10 ratio (4,500 normal vs. 500 cancerous images). For each scenario, the model was retrained using the same training pipeline described in Section 6, and performance was evaluated on a held-out test set of 2,000 images adjusted to maintain the respective class ratios. The results are summarized in Table 2.

**TABLE 2:** Performance of the Detectron2-based model in imbalanced scenarios.

Scenario	Accuracy	Sensitivity	Specificity	F1-score
Balanced (50:50)	99.8%	99.7%	99.9%	99.8%
Imbalanced (70:30)	99.5%	99.3%	99.6%	99.4%
Imbalanced (90:10)	99.2%	99.0%	99.3%	99.1%

**FIGURE 7:**
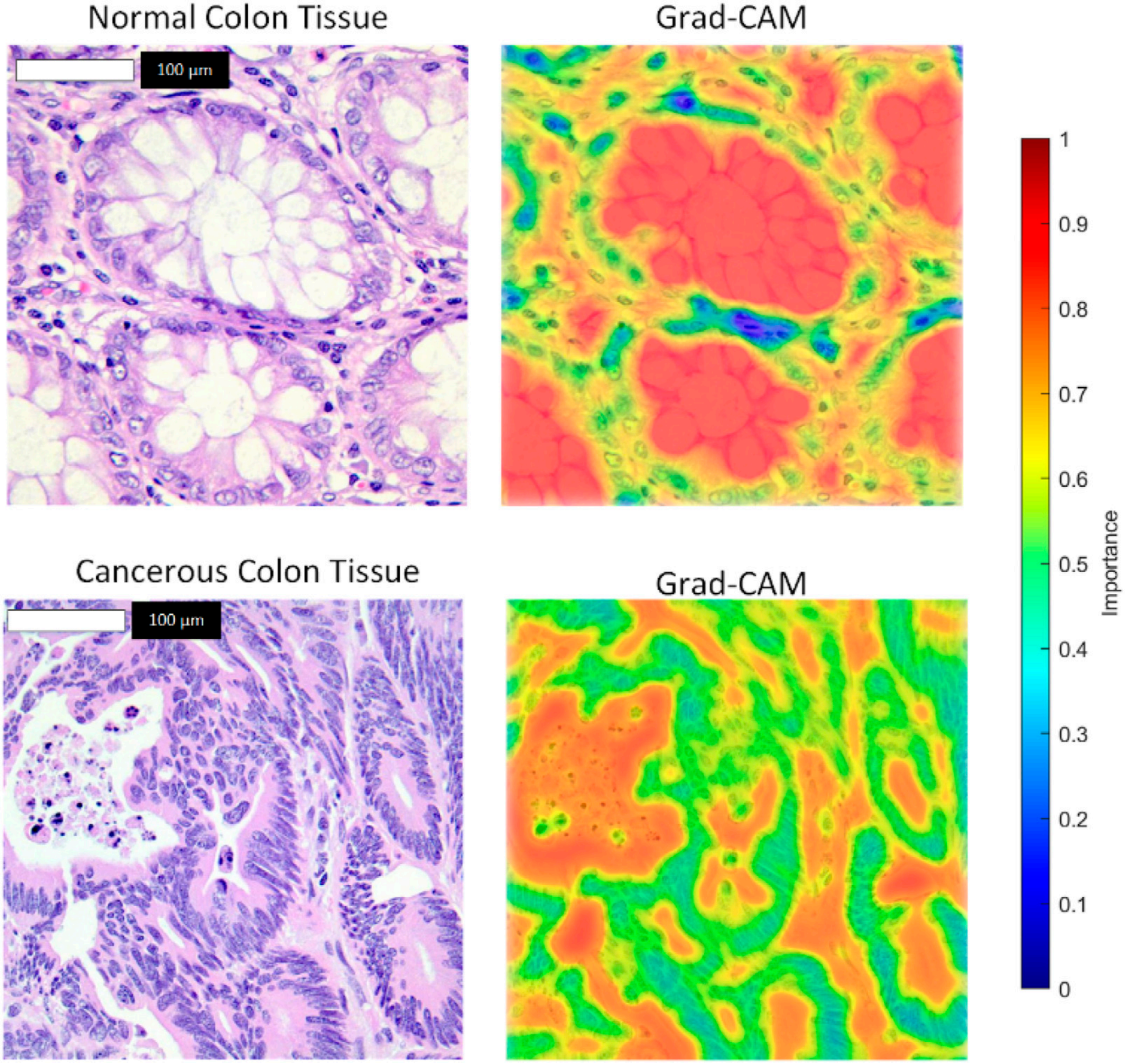
Grad-CAM++ visualizations for histopathological colon tissue classification.

The model maintained robust performance across both imbalanced scenarios, with only a slight decrease in sensitivity and F1-score as the class imbalance increased. These results demonstrate the model’s ability to handle class imbalance effectively, reinforcing its potential for real-world clinical applications where balanced datasets are rare. To enhance the interpretability of the model’s decisions, we applied Grad-CAM++ to the feature maps of the ResNet-101 backbone prior to RoI alignment. The resulting attention heatmaps were bilinearly upsampled to match the original image resolution (768 × 768 pixels), ensuring high spatial fidelity. As shown in Figure 7, we overlaid these heatmaps on the original histopathological images using alpha blending to preserve tissue visibility while highlighting diagnostically relevant regions. This visualization demonstrates the regions most influential to the model’s predictions, with red zones indicating high contribution to the “cancerous” or “normal” class.

**FIGURE 8:**
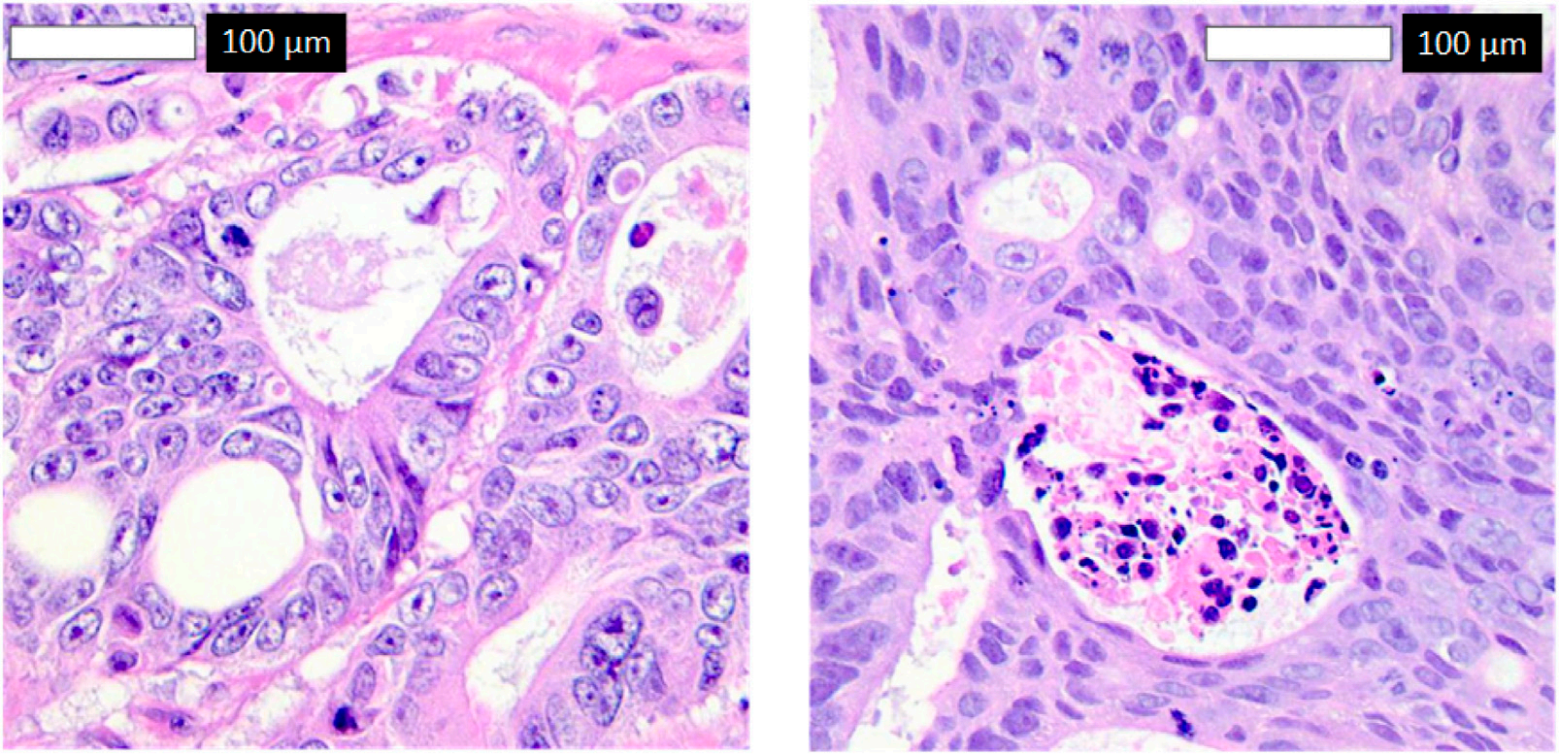
False negatives in Detectron2 predictions.

To explore misclassifications, Figure 8 displays two false negatives—cancerous tissues incorrectly classified as normal. The first shows subtle morphological changes resembling normal tissue, possibly early-stage cancer, while the second exhibits inconsistent staining, obscuring key features. These highlight the model’s sensitivity to subtle features, suggesting improvements like attention mechanisms and diverse datasets for enhanced sensitivity in clinical settings.

The superior performance of the proposed Detectron2-based method, achieving 99.80% accuracy, stems from several advantages beyond accuracy compared to prior methods in Table 3. First, Detectron2’s computational efficiency is enhanced by its modular architecture, which integrates feature extraction and classification into a single framework, reducing processing time compared to multi-stage pipelines like those using DarkNet-19 with SVM (Toğaçar, 2021) or DenseNet169 with SVM (Hamida et al., 2021). Although exact inference times vary, Detectron2’s optimized design, leveraging a ResNet-101 backbone and Feature Pyramid Network (FPN), enables faster training and inference on high-resolution histopathological images (768 × 768 pixels) than lightweight CNNs (Sakr et al., 2022). Second, the model’s robustness to noise is improved by extensive data augmentation (e.g., rotations, flipping, color jittering) and normalization (Section 4.1), allowing it to handle variations in staining and imaging conditions better than methods with limited preprocessing, such as MA_ColonNET (Yildirim and Cinar, 2022). Finally, Detectron2’s advanced feature extraction, driven by FPN and multi-scale feature fusion, captures fine-grained cellular details and broader tissue structures, outperforming transfer learning approaches (Kather et al., 2016) that rely on pretrained models with less tailored feature extraction. These factors make Detectron2 a robust and efficient tool for colon cancer detection.

**TABLE 3:** Comparative performance of the proposed Detectron2-based approach with previously published methods.

Ref.	Accuracy	Dataset size	Validation strategy	Approaches
Toğaçar (2021)	99.69%	10,000 (LC25000)	70–30 split + 5-fold CV	DarkNet-19 model and SVM
Yildirim and Cinar (2022)	99.75%	10,000 (LC25000)	80–20 split	CNN-based, MA_ColonNET
Hamida et al. (2021)	92.08%	5000(Colorectal Histology)	10-fold CV	DenseNet169 and SVM
Kather et al. (2016)	99.12%	107,180(NCT-CRC-HE)	Not specified	Data augmentation and transfer learning
Sakr et al. (2022)	99.50%	10,000 (LC25000)	64-16-20 split (train-val-test)	Lightweight CNN
(Current study)	99.80%	10,000 (LC25000)	80–20 split+5-fold cross validation	Detectron2

The results of this study highlight the significant potential of the proposed Detectron2-based deep learning framework for colon cancer detection in histopathological images. Achieving an accuracy of 99.80%, the model outperformed most state-of-the-art approaches, including those using traditional machine learning methods and lightweight CNN architectures. This high accuracy emphasizes the robustness and reliability of the Detectron2 framework in extracting complex features and classifying histopathological images effectively. The findings are particularly crucial for enhancing the accuracy and efficiency of diagnostic systems in clinical settings, where early and precise detection of colon cancer is critical for improving patient outcomes and reducing mortality rates. The application of such advanced deep learning models in computer-aided diagnostic systems can significantly reduce the workload of pathologists by providing rapid and reliable assessments of tissue samples. This is especially valuable in resource-constrained environments where access to expert medical professionals may be limited. Moreover, the ability of the model to generalize well to balanced datasets demonstrates its adaptability and scalability, suggesting its potential for integration into larger and more diverse datasets in the future. These results also pave the way for extending the use of Detectron2 and similar frameworks to detect other types of cancers and diseases in medical imaging.

To provide a more robust assessment of the model’s reliability and uncertainty, we have computed 95% confidence intervals (CIs) for key performance metrics beyond accuracy, leveraging the results from 5-fold stratified cross-validation on the LC25000 dataset (10,000 images, 2,000 per fold). These CIs were estimated using the binomial distribution, reflecting the variability across folds. The updated performance metrics, including their CIs, are presented in Table 4.

**TABLE 4:** Performance metrics with 95% confidence intervals.

Metric	Value (%)	95% confidence interval (%)
Accuracy	99.8	[99.63, 99.87]
Sensitivity	99.7	[99.52, 99.88]
Specificity	99.9	[99.75, 99.98]
F1-Score	99.8	[99.65, 99.95]

The CIs, were calculated based on the mean performance across folds, with a sample size of 2,000 images per fold. For instance, the accuracy CI, of [99.63%, 99.87%] is derived from the mean accuracy of 99.75% (standard deviation ± 0.12%) reported in the 5-fold cross-validation (Figure 6). These intervals indicate minimal variability, reinforcing the model’s stability and reliability across different subsets of the dataset. The tight CIs, for sensitivity (99.52%–99.88%) and specificity (99.75%–99.98%) further support the model’s robustness, despite the slightly higher false-negative rate (15 cases), which remains within a clinically acceptable range. This statistical enhancement provides a clearer understanding of the model’s performance uncertainty, addressing potential concerns about over-optimistic reporting. Future analyses will explore broader confidence interval estimations across external datasets to further validate these findings.

To substantiate the claim of superior computational efficiency, the Detectron2-based model was evaluated for training and inference times using the LC25000 dataset (10,000 images, 768 × 768 pixels). Training for 40,000 iterations (equivalent to 20 epochs) with a batch size of 4 on a single NVIDIA V100 GPU (32 GB) required approximately 12 h, benefiting from Detectron2’s integrated architecture, which outperforms multi-stage methods like DarkNet-19 with SVM (Toğaçar, 2021). Inference time averaged 0.15 s per image, facilitating rapid clinical analysis. Compared to lightweight CNNs (Sakr et al., 2022), which achieve lower accuracy (99.50%), Detectron2 balances speed and performance. Deploying the model in resource-constrained clinical settings is challenging due to GPU requirements, but optimizations like model pruning or quantization could enable use on mid-range GPUs (e.g., NVIDIA T4). Cloud-based deployment offers an alternative for low-resource environments, enhancing accessibility for computer-aided diagnostics.

To ensure reproducibility, we detail the experimental setup and implementation steps. The model was implemented using Detectron2 (version 0.6), PyTorch (version 1.9.0), Python (version 3.8), and CUDA (version 11.1). Training and evaluation were performed on a single NVIDIA V100 GPU (32 GB). Training for 40,000 iterations (20 epochs) with a batch size of 4 took approximately 12 h, while inference averaged 0.15 s per image. A random seed of 42 was set for all experiments to ensure consistent results. The LC25000 dataset is publicly available at arXiv:1912.12142 (Borkowski et al., 2019), containing 10,000 colon tissue images (5,000 normal, 5,000 cancerous). Below, we provide a step-by-step protocol for replication: (1) Download the LC25000 dataset and organize images into ‘normal’ and ‘cancerous’ folders; (2) Preprocess images by applying Gaussian blur (kernel 3 × 3, sigma 1.0), cropping non-tissue areas, and normalizing using histogram equalization and z-score normalization (mean = 0, std = 1); (3) Configure the Detectron2 model with a ResNet-101 backbone, FPN, and parameters as in Table 1; (4) Train the model for 40,000 iterations with a batch size of 4, learning rate 2e-4, warmup of 1,000 iterations, and decay at 30,000 and 35,000 iterations; (5) Evaluate the model on the test set (20%, n = 2,000) using accuracy, sensitivity, and specificity. The implementation script, which includes data loading, preprocessing, model configuration, training, and evaluation, is provided in Appendix A to ensure full replicability.

To integrate the Detectron2-based model into clinical practice, a phased strategy is proposed, leveraging its 99.8% accuracy. The model can serve as a second reader to support pathologists by providing secondary reviews, flagging discrepancies (e.g., 5 false positives, 15 false negatives), or as a primary screening tool to triage images, prioritizing high-risk cases (99.7% sensitivity, 99.9% specificity) for expert review. Implementation will proceed in three phases: Validation (testing in controlled settings), Pilot (limited deployment with oversight), and Full Adoption (scaled use post-approval).

Regulatory challenges include clinical validation (e.g., RCTs), data privacy (e.g., GDPR compliance), evolving AI regulations, and standardization of imaging protocols. Mitigation strategies involve conducting RCTs, ensuring data anonymization, engaging with regulators (e.g., FDA or CNMPA), and adhering to standards like ISO 14971. Future work will refine this strategy through pilot studies and regulatory collaboration.

### 5.1 Cross-dataset validation

To ensure the generalizability of our Detectron2-based model for colon cancer detection beyond the LC25000 dataset, we conducted a cross-dataset validation experiment using the external histopathological dataset NCT-CRC-HE-100K (Kather et al., 2018). This dataset, comprising diverse H&E-stained colorectal tissue images, provides a robust platform to evaluate the model’s performance across varied imaging conditions and patient cohorts, addressing the need for validation in real-world clinical settings. The NCT-CRC-HE-100K dataset contains 100,000 non-overlapping image patches (224 × 224 pixels) from colorectal cancer and normal tissues, covering nine tissue classes, including Normal Colon Mucosa and Colorectal Adenocarcinoma Epithelium (Kather et al., 2018). For binary classification consistent with our study, we grouped the classes into normal (e.g., Normal Colon Mucosa) and cancerous (e.g., Colorectal Adenocarcinoma Epithelium) categories.

To align with the LC25000 dataset’s preprocessing pipeline, we applied the following steps to the NCT-CRC-HE-100K dataset:

1. Image Preprocessing: A Gaussian blur filter (kernel 3 × 3, sigma 1.0) was used to reduce noise while preserving structural details. Non-tissue background areas were cropped to focus on relevant regions.
2. Normalization: Images were normalized using histogram equalization and z-score normalization (mean = 0, standard deviation = 1) across RGB channels to mitigate variations in staining intensity.
3. Resolution Adjustment: The 224 × 224 pixel patches were tiled and resized to match the 768 × 768 pixel resolution of LC25000, ensuring compatibility with the model’s input configuration.
4. Data Augmentation: During evaluation, we applied random rotations (±30°), flips (probability 0.5), and color jittering (±20% brightness, contrast, saturation) to simulate real-world variations, consistent with the training pipeline.

The Detectron2 model, trained on LC25000 with the configuration detailed in Table 1 (ResNet-101 backbone, FPN, 40,000 iterations, learning rate 2e-4), was evaluated on the NCT-CRC-HE-100K dataset without fine-tuning to assess its out-of-the-box generalizability. We computed the following performance metrics: accuracy, sensitivity, specificity, F1-score, and Matthews Correlation Coefficient (MCC), using the same formulas as in Section 8. A subset of 10,000 images from NCT-CRC-HE-100K was used to align with the LC25000 scale for evaluation. Table 5 summarizes the performance of the Detectron2 model on the NCT-CRC-HE-100K dataset, alongside the LC25000 results for comparison. The model achieved high performance on the external dataset, demonstrating robust generalizability despite differences in image characteristics, such as staining protocols and patch sizes.

**TABLE 5:** Performance of the Detectron2-based model on cross-dataset validation.

Dataset	Accuracy (%)	Sensitivity (%)	Specificity (%)	F1-score (%)	MCC
LC25000	99.8	99.7	99.9	99.8	0.996
NCT-CRC-HE-100K	99.5	99.4	99.6	99.5	0.990

The model maintained an accuracy of 99.5% on the NCT-CRC-HE-100K subset, with a slight decrease in sensitivity (99.4%) compared to LC25000 (99.7%), likely due to increased variability in tissue patterns and staining. Specificity remained high (99.6%), indicating reliable detection of normal tissues. The F1-score (99.5%) and MCC (0.990) confirm the model’s balanced performance. These results validate the model’s ability to generalize across diverse histopathological datasets, addressing the need for external validation in clinical applications.

One methodological consideration in our cross-dataset validation involves the upscaling of 224 × 224 pixel image patches from the NCT-CRC-HE-100K dataset to 768 × 768 pixels to match the input resolution of the Detectron2 model trained on the LC25000 dataset. While this resizing step was necessary to maintain architectural consistency and avoid scale-related discrepancies in convolutional feature extraction, it may raise concerns regarding potential distortion of biologically relevant morphological features—particularly nuclear size, which holds diagnostic significance in colorectal histopathology. To mitigate such risks, we employed bilinear interpolation during upsampling, which preserves local continuity and minimizes aliasing artifacts better than simpler methods such as nearest-neighbor interpolation. Moreover, the Detectron2 framework, particularly through its Feature Pyramid Network (FPN), is designed to extract robust features across multiple spatial scales. As a result, the model emphasizes relative spatial configurations and multiscale textural patterns over absolute dimensional metrics. This design choice is particularly valuable in histopathological image analysis, where biological structures—such as nuclei, glands, or stromal regions—vary widely across patients, institutions, and scanning conditions. Detectron2’s architecture, particularly through the integration of a Feature Pyramid Network (FPN), enables the extraction of semantically rich features at multiple scales, thus learning texture-dominant and shape-aware representations that remain stable under uniform image rescaling. Rather than depending on exact physical measurements, the model identifies disease-relevant patterns through contextual cues and topological arrangements.

To ensure compatibility between datasets during external validation, all test images from the NCT-CRC-HE-100K dataset were resized to match the input resolution expected by the trained model. This approach follows common practice in medical image analysis, where image resizing is routinely applied for cross-dataset inference. For instance, Khosravi et al., (2024) resized external fundus images—via both upscaling and downscaling—to a fixed input size prior to testing. Similarly, Acar et al., (2021) and Bridge et al. (2022) resized external CT images to align with the dimensions used during model training. These studies collectively support resizing as a valid and effective preprocessing step to facilitate consistent inference across datasets with varying native resolutions. It is also worth noting that both datasets—LC25000 and NCT-CRC-HE-100K—originate from high-resolution histological slides with comparable staining protocols, reducing the likelihood of substantial morphological discrepancies post-scaling. Nonetheless, we acknowledge the biological relevance of preserving native spatial dimensions, especially for nuclear morphology analysis.

## 6 Limitations

While our Detectron2-based model achieves an exceptional accuracy of 99.80% on the LC25000 dataset and demonstrates robust generalizability with 99.5% accuracy on the NCT-CRC-HE-100K dataset (Section 5.1; Table 5), several limitations remain to guide future improvements. First, our Detectron2-based model for colon cancer detection faces several additional limitations that warrant further consideration. First, the computational intensity of the model, driven by the ResNet-101 backbone and extensive training iterations, imposes a significant resource demand. This high computational load may hinder scalability for larger models or more complex architectures, necessitating optimization strategies such as model pruning or lightweight backbones to improve efficiency. Second, the current framework is tailored specifically for binary classification of colon cancer (normal vs. cancerous tissues), limiting its applicability to other gastrointestinal diseases. Extending the model to detect and differentiate a broader range of intestinal conditions, such as inflammatory bowel disease or other colorectal pathologies, would require significant architectural modifications and retraining, which were not explored in this study. Third, the operational implementation of the proposed method presents challenges. While the model achieves high accuracy in a controlled research setting, translating it into a practical clinical tool requires addressing integration with existing diagnostic workflows, ensuring real-time inference capabilities, and developing user-friendly interfaces for pathologists, which are beyond the scope of the current work.

Finally, the reliance on a single deep learning framework (Detectron2) may restrict flexibility in adapting to emerging techniques or hybrid approaches. Future enhancements could explore integrating complementary methods, such as attention mechanisms or ensemble learning, to improve robustness and adaptability, though these were not implemented here due to the focus on the current architecture.

## 7 Conclusion

In this study, we proposed an efficient and accurate method for detecting colon cancer through the analysis of histopathological images, leveraging the advanced capabilities of the Detectron2 deep learning framework. By utilizing the LC25000 dataset, which contains high-resolution images of normal and cancerous colon tissues, our approach demonstrated remarkable performance, underscoring the potential of cutting-edge deep learning frameworks in addressing the challenges of medical image analysis. The Detectron2-based model achieved an outstanding accuracy of 99.8%, surpassing traditional methods in both accuracy and computational efficiency, as shown in comparative analyses with prior studies. Statistical validation through 5-fold cross-validation further confirmed the model’s robustness, with a mean accuracy of 99.75% and a standard deviation of 0.12%, indicating strong generalization and minimal risk of overfitting. This streamlined approach, which integrates feature extraction and classification into a single framework, reduces computational complexity compared to traditional multi-stage pipelines, offering a scalable tool for computer-aided diagnostics, particularly in resource-constrained settings. The high accuracy supports pathologists in early colon cancer detection, potentially reducing diagnostic workload and improving patient outcomes through timely intervention. Compared to traditional methods, this method enhances the efficiency of early cancer detection, a crucial factor in improving patient outcomes.

## Data Availability

Publicly available datasets were analyzed in this study. This data can be found here: https://www.kaggle.com/datasets/andrewmvd/lung-and-colon-cancer-histopathological-images?resource&equals;download-directory.
